# Diagnostic challenges in a patient with an occult insulinoma:^68 ^Ga‐DOTA‐exendin‐4 PET/CT and ^68^Ga‐DOTATATE PET/CT

**DOI:** 10.1002/ccr3.1448

**Published:** 2018-03-01

**Authors:** Elisa Bongetti, Melissa H. Lee, David A Pattison, Rodney J. Hicks, Richard Norris, Nirupa Sachithanandan, Richard J MacIsaac

**Affiliations:** ^1^ Department of Endocrinology & Diabetes St Vincent's Hospital Melbourne Victoria Australia; ^2^ Centre for Cancer Imaging Peter MacCallum Cancer Centre Melbourne Victoria Australia; ^3^ Department of Nuclear Medicine & Specialised PET Services Royal Brisbane & Women's Hospital Brisbane Queensland Australia; ^4^ Neuroendocrine Service Peter MacCallum Cancer Centre Melbourne Victoria Australia; ^5^ Department of Pathology St Vincent's Hospital Melbourne Victoria Australia; ^6^ Department of Medicine University of Melbourne Melbourne Victoria Australia

**Keywords:** adenoma, diagnostic imaging, endocrine gland neoplasms, insulinoma, Islet cell, molecular imaging, nesidioblastosis, pancreatic neoplasms

## Abstract

Despite growing evidence for GLP‐1R molecular‐based imaging, successful localization of insulinomas may require the use of multiple imaging modalities. Not all benign insulinomas express the GLP‐1R as expected. Our case demonstrates that there is a still an important role for traditional methods for the anatomical localization of an insulinoma.

## Case history

An 82‐year‐old woman with hyperinsulinemic hypoglycemia had glucagon‐like peptide 1 receptor (GLP‐1R) and somatostatin receptor subtype 2 (SSTR2)‐based functional imaging, but these scans failed to localize a pancreatic insulinoma. While recent studies of GLP‐1R‐based functional imaging demonstrate high accuracy for insulinoma localization, this case demonstrates potential limitations.

An 82‐year‐old woman presented with symptomatic hypoglycemia (blood glucose level (BGL) 2.4 mmol/L), which was corrected to 6.8 mmol/L with intravenous dextrose. She reported a three‐year history of similar episodes with neuroglycopenic and autonomic symptoms, such as dizzy spells, hunger, confusion, and visual changes while fasting, which were relieved by consuming glucose‐rich foods.

On examination, her blood pressure was 130/60 mmHg, and her heart rate was 90 beats per minute and oxygen saturation 98% on room air. Her abdomen was tender to palpation in the right upper quadrant; however, the rest of her abdomen was soft with no palpable masses. Examination of cardiorespiratory and neurological systems was unremarkable. The patient's past history included gastroesophageal reflux disease, hypertension, and hypercholesterolemia. Her regular medications included simvastatin and esomeprazole. She had no history of diabetes mellitus or other endocrinopathies and no previous gastric surgery.

## Investigations

Seventy‐two‐hour fast elicited a symptomatic hypoglycemic episode (BGL 1.7 mmol/L) associated with elevated serum insulin and C‐peptide levels (12 mU/L [<3 mU/L] and 1.31 pmol/mL [≤0.20 pmol/mL], respectively) consistent with hyperinsulinemic hypoglycemia. Sulfonylurea screen and insulin antibodies were negative. Appropriate counter‐regulatory hormonal response was also documented, and her cortisol peak (749 nmol/L) during a short Synacthen test was within normal limits. Thyroid‐stimulating hormone and human growth hormone were within normal limits (1.58 *μ*U/mL [0.35–4.94 *μ*U/mL] 9 nmol/L [7–22 nmol/L], respectively).

Triple‐phase CT scan of the pancreas showed a bulky pancreatic body but no distinct pancreatic mass. ^68^Ga‐DOTA‐octreotate PET/CT was reported as negative for somatostatin receptor avid insulinoma. The patient proceeded to have glucagon‐like peptide 1 receptor‐based imaging with ^68^Ga‐DOTA‐exendin‐4 PET/CT. This scan revealed diffuse uptake throughout the pancreas with a maximum standardized uptake value (SUVmax) of 6.3, which was felt to be suggestive of nesidioblastosis in this clinical context.

Our patient required extended inpatient hospitalization for persistent hypoglycemic episodes. Attempts were made to optimize her medical management using 1 mg dexamethasone at night and small, frequent, complex carbohydrate meals. Nevertheless, her hypoglycemic episodes continued and she required overnight dextrose infusions.

Despite the results of the above scan, there was still a high clinical suspicion of insulinoma with recurrent fasting hypoglycemia. Further investigation with a selective arterial calcium stimulation test (SACST) with hepatic venous sampling was therefore undertaken (see Table 1). This demonstrated a greater than twofold increase in insulin levels in the proximal and distal splenic arteries and the gastroduodenal artery suggesting the presence of a hyperfunctional lesion in the body and/or tail region of the pancreas. The patient was subsequently commenced on diazoxide 50 mg three times daily in addition to dexamethasone to help manage her neuroglycopenic symptoms.

**Table 1:** Selective intra‐arterial calcium stimulation test: insulin levels (mU/L). Normal insulin <10 mU/L

Artery	Time (mins)					
	−120	0	+30	+60	+90	+120
Proximal splenic artery	3	10	**42**	**62**	**38**	**22**
Distal Splenic artery	3	4	24	21	15	11
Common hepatic artery		11	9	10	15	17
Gastroduodenal artery	10	12	18	22	22	22
Superior mesenteric artery		12	12	14	12	17

Evidence below of abnormally elevated levels of insulin predominantly in proximal splenic artery shown in bold.

## Management

To further delineate the site of the lesion, the patient underwent endoscopic ultrasound (EUS) which localized a lesion (11 x 6 mm) at the junction of the pancreatic body and tail. Fine needle aspirate obtained at the time of EUS confirmed the presence of an islet cell tumor. The patient underwent laparoscopic enucleation of the lesion and sampling of adjacent pancreatic tissue. Histology confirmed an insulinoma with positive immunohistochemistry for chromogranin, synaptophysin, and insulin. Adjacent pancreatic tissue was normal with no evidence for nesidioblastosis. Immunohistochemistry demonstrated the insulinoma was negative for GLP‐1R but strongly positive for SSRT2.

**Figure 1:**
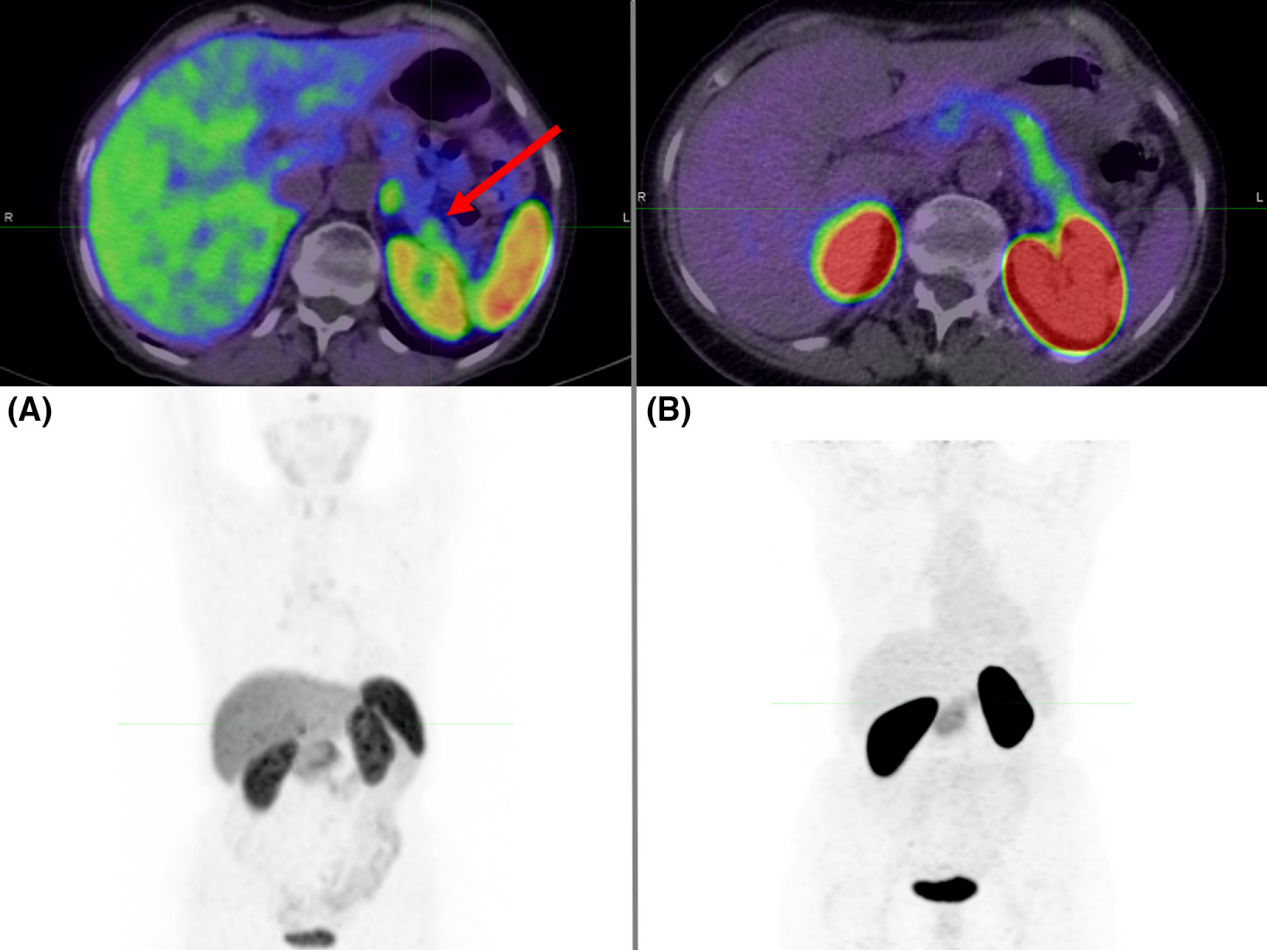
(A) ^68^Ga‐DOTATATE PET/CT. Mild focal uptake in pancreatic tail suspicious for insulinoma but difficult to delineate from adjacent renal activity. (B) ^68^Ga‐DOTA‐exendin‐4 PET/CT demonstrates diffuse pancreatic uptake higher than what would be expected physiologically, suggestive of nesidioblastosis without focal intense uptake to indicate insulinoma. Again, intense renal uptake potentially compromises assessment of the pancreatic tail.

Given the above results, a subsequent retrospective review of the ^68^Ga‐DOTA‐octreotate PET/CT by nuclear medicine specialists at a quaternary neuroendocrine tumor (NET) referral service identified a subtle lesion suspicious for insulinoma in the tail of the pancreas (Figure 1), which had not been identified at the time of initial diagnostic workup due to its proximity to high uptake in the left kidney.

## Outcome and Follow‐up

There were no intraoperative or postoperative complications. In particular, blood glucose levels were stable during the operation and rose to 14 mmol/L immediately postoperatively, and thereafter remained within the normal range. The patient was followed up in an endocrinology outpatient clinic within 1 week of her surgery. She reported complete resolution of her symptoms with BSLs ranging between 6 and 7 mmol/L. Her blood pressure was 155/80 mmHg, and she denied dizziness, nausea, vomiting, or headaches suggestive of cortisol deficiency. Her diazoxide was ceased, and her dexamethasone was successfully weaned. She had ongoing complete resolution of symptoms over the subsequent 12 months follow‐up in endocrinology clinic. There was no evidence of insulinoma recurrence on CT of the abdomen/pelvis with IV contrast approximately 6 months postoperatively.

## Discussion

Insulinomas are rare neuroendocrine tumors most commonly located in the pancreas. They are the most frequent cause of hyperinsulinemic hypoglycemia in adults without diabetes, and the majority (90%) are benign 1. The so‐called rule of 10 states that 10% of insulinomas are multiple, 10% are malignant, 10% are associated with multiple endocrine neoplasia type 1 (MEN1), and 10% are ectopic 2. Bariatric surgeries are increasingly common procedures that can be complicated by late dumping syndrome, which is postprandial hyperinsulinemic hypoglycemia 3. It is important to make a differential between late dumping syndrome and insulinoma. Clinical and biochemical diagnosis of insulinoma is generally followed by radiological localization of the lesion. Surgical excision offers the only potential cure 4. Localization of the lesion may be challenging as insulinomas are typically solitary and less than two centimeters in diameter 4.

The sensitivity for detecting insulinomas has been reported to be approximately 75% for CT and 55–90% with MRI imaging 5. EUS has been reported to have excellent sensitivity (85–95%); however, insulinomas are often located in regions of the pancreas which makes them more difficult to visualize 6, 7. SACST is the most invasive localizing procedure, however has been reported as the most sensitive modality (95–100%) 2. Other investigation modalities such as intraoperative ultrasound and manual palpation of the pancreas are reported to have sensitivities of 80–99% and 75–95%, respectively 1.

Recently, there has been a growing body of literature supporting the efficacy of GLP‐1R PET/CT imaging for insulinomas based upon the near ubiquitous GLP‐1R expression on beta cells 5, 6. Immunohistochemistry staining for GLP‐1R on the insulinoma in our patient was, however, negative. There have been other reports of variation in the expression of GLP‐1R and SSRT2 on benign insulinomas 1, 8. It has been suggested that insulinomas can be investigated sequentially with GLP‐1R‐ and SSTR2‐based molecular imaging because all insulinomas would be expected to express either one or both of these receptors 1, 8. In a prospective cohort study of patients with histopathologically confirmed insulinomas, ^68^Ga‐NOTA‐exendin‐4 GLP‐1R PET/CT correctly detected insulinomas in 42 of 43 (97.7%) patients 1. The single false‐negative case was negative for GLP‐1R expression but avid for SSTR2 on somatostatin receptor scintigraphy similar to the current case.

A recent review described the relationship of molecular markers with malignancy in insulinomas as the “triple‐flop” phenomenon, representing an increasing tendency toward malignancy during progression from GLP‐1R avid, to somatostatin receptor (SSTR) avid, to 2‐[18F]Fluoro‐2‐Deoxy‐D‐Glucose (FDG) avid insulinomas 8. The SSTR‐positive and GLP‐1‐negative nature of this case raises the possibility that our patient may have been at risk of transformation to malignancy and requires ongoing surveillance.

In this case, GLP‐1R PET/CT was suggestive of nesidioblastosis. However, nesidioblastosis was inconsistent with her history of fasting (rather than postprandial) hypoglycemia and histological findings of normal pancreatic tissue adjacent to the insulinoma. The intensity of uptake (SUVmax 6.3) on the GLP‐1R PET/CT was similar to a case report of histopathologically proven nesidioblastosis (SUVmax 6.9) 9. The authors of this report suggested a role for GLP‐1R PET/CT in preoperative assessment when differentiating nesidioblastosis from insulinoma 9. Our case suggests that further research involving a larger case series is needed before the value of a GLP‐1R PET/CT can be recommended for preoperative assessment.

^68^Ga‐DOTATATE PET/CT was initially reported as negative despite subsequent immunohistochemical analysis demonstrating positive SSTR2 expression. However, retrospective review by nuclear medicine physicians with a special interest in neuroendocrine imaging subsequently identified a suspicious lesion with focal uptake subtly greater than adjacent tissue within the pancreatic tail. This highlights the variable molecular imaging phenotype of insulinoma (typically localized on either GLP‐1R or ^68^Ga‐DOTATATE PET/CT, but not both), and we recommend that when practical, these specialized scans be performed in centers with expertise in neuroendocrine imaging given the potential challenges in their interpretation. Lesions in the tail of the pancreas, which can be immediately adjacent to the left kidney, may also be masked by intense uptake in the latter, which likely compromised detection in this case.

## Conclusion

In summary, localization of insulinomas is challenging and may require the use of multiple imaging modalities and specialized expertise. While recent studies of GLP‐1R and SSTR2 molecular‐based imaging demonstrate potentially high accuracy for insulinoma localization, our case demonstrates that there is still an important role for traditional methods for the anatomical localization of an insulinoma in cases where noninvasive imaging fails to localize a lesion. We also recommend that molecular imaging of these rare tumors be performed in specialized centers and that traditional methods, such as EUS and SACST, not be abandoned prematurely.

## Authorship

EB: main author of this study. MHL: provided insights into the case history and contributed to creating and revising report. DAP: assisted in revisions and offered expertise within the field of endocrinology, nuclear medicine, and neuroendocrine tumors. RJH: assisted in revisions and offered expertise within the field of nuclear medicine and neuroendocrine tumors. RN: offered expertise within the field of pathology to re‐examine samples and clarify findings. NS: assisted in revisions and offered expertise within the field of endocrinology. RJM: oversaw editing and offered expertise within the field of endocrinology.

## Conflict of Interest

None declared.
